# Characterizing Longitudinal Changes in the Impedance Spectra of In-Vivo Peripheral Nerve Electrodes

**DOI:** 10.3390/mi9110587

**Published:** 2018-11-12

**Authors:** Malgorzata M. Straka, Benjamin Shafer, Srikanth Vasudevan, Cristin Welle, Loren Rieth

**Affiliations:** 1 Center for Bioelectronic Medicine, Feinstein Institute for Medical Research, Northwell Health, Manhasset, NY 11030, USA; lrieth@northwell.edu; 2 U.S. Food and Drug Administration, Center for Devices and Radiological Health (CDRH), Office of Science and Engineering Laboratory (OSEL), Division of Biomedical Physics (DBP), Silver Spring, MD 20993, USA; benshafer92@gmail.com (B.S.); Srikanth.Vasudevan@fda.hhs.gov (S.V.); 3 Departments of Neurosurgery and Bioengineering, University of Colorado, Aurora, CO 80045, USA; cristin.welle@ucdenver.edu; 4 Departments of Electrical Engineering and Bioengineering, University of Utah, Salt Lake City, UT 84112, USA

**Keywords:** impedance, Utah electrode arrays, electrode–tissue interface, peripheral nerves

## Abstract

Characterizing the aging processes of electrodes in vivo is essential in order to elucidate the changes of the electrode–tissue interface and the device. However, commonly used impedance measurements at 1 kHz are insufficient for determining electrode viability, with measurements being prone to false positives. We implanted cohorts of five iridium oxide (IrOx) and six platinum (Pt) Utah arrays into the sciatic nerve of rats, and collected the electrochemical impedance spectroscopy (EIS) up to 12 weeks or until array failure. We developed a method to classify the shapes of the magnitude and phase spectra, and correlated the classifications to circuit models and electrochemical processes at the interface likely responsible. We found categories of EIS characteristic of iridium oxide tip metallization, platinum tip metallization, tip metal degradation, encapsulation degradation, and wire breakage in the lead. We also fitted the impedance spectra as features to a fine-Gaussian support vector machine (SVM) algorithm for both IrOx and Pt tipped arrays, with a prediction accuracy for categories of 95% and 99%, respectively. Together, this suggests that these simple and computationally efficient algorithms are sufficient to explain the majority of variance across a wide range of EIS data describing Utah arrays. These categories were assessed over time, providing insights into the degradation and failure mechanisms for both the electrode–tissue interface and wire bundle. Methods developed in this study will allow for a better understanding of how EIS can characterize the physical changes to electrodes in vivo.

## 1. Introduction

Impedance measurements are one of the most widely used techniques to evaluate neural electrodes, both on the benchtop and in vivo. Single frequency impedance measures (e.g., 1 kHz) can be obtained rapidly, used to diagnose open and short circuit failures, and confirm that impedances are compatible with electrical stimulation. Impedance measurements have the possibility of conveying information regarding other key factors of electrode performance, including the (1) degradation of neural electrodes, (2) changes in the electrode–tissue interface, and a (3) correlation to the quality of the acquired neural data. However, the direct interpretation of impedance changes of Utah arrays has remained elusive, despite the development of sophisticated impedance models [1,2]. This is, in part, due to the complexity of the external circuit, compounded by the limited range of the frequencies collected to form the impedance spectra, as well as the compromises in the impedance measurement methods. In addition, for microelectrode arrays such as the Utah electrode array (UEA), abiotic and biotic changes at the electrode tissue interface can drive both increases and decreases in impedance, such that the competition between these mechanisms can result in complex changes in impedance over time [3]. The impedance changes due to abiotic sources are from the changes in the material and surface area of the electrode, whereas the biotic changes result from alterations in the tissue and physiological environment between the electrode and the counter electrode. Moreover, even profound physical damage, such as broken lead wires, can sometimes result in fairly modest changes in the measured 1 kHz impedance. Circuit models for these individual changes have been thoroughly discussed in the literature [1,2]. However, the diversity of the factors that influence impedance, often simultaneously in opposite valences, makes the characterization of the electrode/tissue interface and the identification of failure modes complex. This work combines elements of several models [1,2,4], and enables the categorization of spectra from Utah arrays based on common characteristics.

The sheer volume of data generated by repeated broad-spectrum EIS measurements from high channel-count interfaces also adds to the difficult process of interpreting data. As part of a larger effort to investigate the failure mechanisms of Utah arrays in-vivo, this work was intended to provide a resource-efficient method so as to characterize impedance dynamics and pinpoint failure mechanisms. With this aim, impedance spectra were collected from 1 to 10^6^ Hz, from 16 channel UEAs implanted in the sciatic nerve relative to platinum/iridium reference (aka counter or ground) wires in the adjacent tissue of rat models (*N* = 11), for up to 12 weeks. The spectra were classified using an algorithm that we developed to distinguish commonly observed trends in the magnitude and phase spectra. The results of this classification were supervised by our team, and on rare occasions, the classification was manually corrected. Both the performance of the classification, its impact on error analysis in aggregated spectra, and its representation of long-term trends were analyzed. Portions of the spectra up to 10^4^ Hz were fitted with a simple Randles circuit model to quantify their electrical characteristics, and to perform quantitative statistical comparisons between them. Furthermore, these fits provided additional insight in to changes of the circuit over time, as well as likely failure modes.

These results provide a high-throughput approach to quantifying changes in impedance spectra under in-vivo conditions. The algorithm significantly increases the ability to diagnose specific failure modes, both biotic and abiotic, in order to provide insight into the real-time integrity of the implanted electrode and biological response.

## 2. Materials and Methods

The surgical implantation and data collection of the impedance spectra occurred at the Food and Drug Administration. This study was approved by the Institutional Animal Care and Use Committee (IACUC) at the Food and Drug Administration, White Oak campus (protocol number WO2014-145, approved April 2014). Female Lewis rats were purchased from Charles River Laboratories International Inc., for the experiments described in this manuscript. The experiments were conducted on animals weighing between 200 and 280 g at the time of surgery. The animals were single housed in plastic cages with 12-h light and dark cycles throughout the experiments. The animals were randomly assigned to one of the following two groups: (1) IrOx Utah arrays (*N* = 5) and (2) Pt Utah arrays (*N* = 6). Six iridium oxide arrays were originally planned for the study, but one broke during surgical implantation and was removed from the data analysis. Impedance data was collected as part of a different study for evaluating nerve and electromyography (EMG) activity (not presented here).

**Figure 1:**
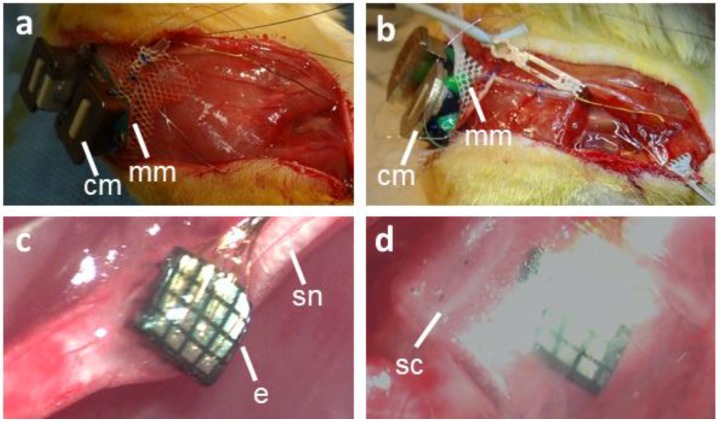
Surgical procedure for the implantation of a nerve electrode array. (**a**) Bronze infused stainless-steel connector mount secured to the lumbar fascia. (**b**) Two-part connector mount secured to the lumbar fascia. (**c**) Electrode array implanted into the sciatic nerve. (**d**) Silicone cuff used to secure the electrode array inside the sciatic nerve. cm—connector mount; mm—mersilene mesh, e—electrode array, sn—sciatic nerve; sc—silicone cuff.

Sixteen channel (16 recording) Blackrock microelectrode arrays with shanks arranged in a 4 × 4 configuration were used in this study. Each shank was 1 mm in length with a 0.4 mm pitch between electrodes. The Utah arrays with platinum electrode contacts and Parylene-C insulation were purchased from Blackrock Microsystems. The Iridium Oxide Utah electrode arrays (UEAs), which had a low impedance tip metallization relative to Pt, were provided by Rohit Sharma, Ryan Caldwell, Brian Baker, and Dr. Loren Rieth. The production of these arrays has been described previously [5,6,7]. Briefly, iridium oxide arrays were fabricated from *p*-type silicon with trenches diced into the backside, and were back-filled with glass for electrical isolation. Subsequently, the front side was diced to the glass and the resulting shanks were etched to form needle-shaped electrodes. The backside bond pads were sputter-deposited from Pt and Ir, and the frontside metallization was sputter-deposited from Pt/Ir/IrOx. The electrodes were encapsulated with 6 µm of Parylene-C and the tips were de-insulated using an oxygen plasma process. The electrodes were wire-bonded to the circuit-board and the bond pads were encapsulated using silicone. The electrodes and the connector (A79026-001, Omnetics Connector Corporation, Minneapolis, MN, USA) were linked via a 55 mm long lead wire bundle, as shown in Figure 1a,b. A 2.5 cm long silicone tube starting at the connector level was added to protect the leads wires from high mechanical stress. Custom-designed EMG arrays were purchased from Microprobes for Life Science, with eight insulated stainless-steel wires of 110 mm length and 0.1 mm diameter arranged into four bipolar pairs, attached to an Omnetics connector (A79038-001) (not shown).

The connector mounts were designed using SolidWorks (2014, Dassault Systèmes SolidWorks Corp., Waltham, MA, USA). Two types of connector mounts were used in this study, the first of which was 3D printed with bronze infused stainless-steel (Figure 1a), which was used in two Pt Utah arrays. The second mount had the bottom part 3D printed with Nylon and the top part with bronze infused stainless steel (Shapeways.com) (Figure 1b). A 35 mm × 30 mm Mersiline mesh was attached to the bottom part of the connector mount using epoxy (Loctite Hysol Epoxy). Connectors from the nerve microelectrode array (and the EMG electrode array were inserted into their respective places inside the connector mount and were bonded with epoxy and, finally, Kwik-cast (World Precision Instruments, Sarasota, FL, USA) were thoroughly applied to the bottom portion of the connector mount to provide a softer tissue contacting surface.

### 2.1. Surgical Procedure

The animals were anesthetized with an intraperitoneal injection of a ketamine (75 mg·kg^−1^) and Dexmedetomidine (0.25 mg·kg^−1^) cocktail. After confirming the lack of a toe pinch reflex, the surgical site was shaved starting from the thoracic region down to the right leg, and was sterilized with betadine and alcohol. An ocular lubricant was applied to prevent drying, and 3 mL of warm sterile saline was administered subcutaneously to prevent dehydration.

A skin incision was made over the thoracic/upper lumbar fascia, and which extended to the right biceps femoris. The connector mount was secured to the fascia with sutures (4-0, Prolene, Polypropylene suture, Med-Vet International, Mettawa, IL, USA), as shown in Figure 1a,b. Mersilene mesh was gently tucked under the skin after dissecting the underlying connective tissue. The sciatic nerve was exposed using blunt dissection techniques described elsewhere [8,9,10]. The nerve was freed from the surrounding connective tissue, and a piece of sterile silicone block (~10 mm × 8 mm × 1 mm sylgard 184 silicone elastomer kit, Dow Corning, Midland, MI, USA) was placed underneath the nerve. A small sheet of sterile parafilm was placed between the nerve and the silicone block. The UEA array was carefully positioned over the nerve and inserted using a pneumatic impactor (Blackrock Microsystems, Salt Lake City, UT, USA) set at 10 psi. The electrode was gently tapped with the impactor ~5–6 times, so as to achieve insertion. Once the shanks were inside the nerve, a few drops of a two-part fibrin sealant (TISSEEL, Baxter Healthcare Corporation, Deerfield, IL, USA) were applied to stabilize the construct, as shown in Figure 1c. After the fibrin sealant was cured, the silicone block and parafilm were removed from underneath the nerve, followed by the placement of a 4 mm long, 3.2 mm inner diameter silicone cuff around the nerve and electrode. The lumen of this silicone tube was filled with a fibrin sealant, as shown in Figure 1d (cloudy inside silicone tube). The muscle incision over the nerve was closed. The delaminated ground and reference leads were threaded and secured at two different locations inside the muscle over the sciatic nerve implant site using a 21 G needle serving as a cannula.

To implant the EMG arrays, a skin incision was made between the right gastrocnemius and the tibialis anterior muscle. EMG wires were tunneled under the skin, and two pairs of EMG wires for the gastrocnemius and two pairs for the tibialis anterior were inserted into the muscles for redundancy using a 25 G needle serving as a cannula. The wires were then secured in place using sutures (4-0, Prolene, OASIS). Finally, all of the skin incisions were closed using a combination of sutures (4-0, Prolene, OASIS) and the application of gluture. The animals were then administered Atipamezole (0.5 mg kg^−1^, i.p. (intraperitoneal)) for anesthesia reversal, Meloxicam (2 mg kg^−1^, s.c. (subcutaneous)) for analgesia, and Gentamycin (8 mg kg^−1^, s.c.) for antibiotic treatment. Meloxicam (1 mg kg^−1^, s.c.) per day for two days was administered following surgery.

### 2.2. Impedance Measurements

The broadband impedance measurements of the individual channels with respect to the ground wire were recorded using electrochemical impedance spectroscopy (1–10^6^ Hz, Gamry Instruments Inc., Warminster, PA, USA). To record the pre-implant measurements of a given channel, the electrode array and the ground wires were immersed in 0.15 M phosphate buffered saline. For the post-implant measurements, the impedance of the arrays was measured immediately (~1 h) after the surgery, to confirm the device function, and again, weekly, starting two weeks, for 12 weeks or until device failure.

### 2.3. Visualization of Spectra

**Figure 2:**
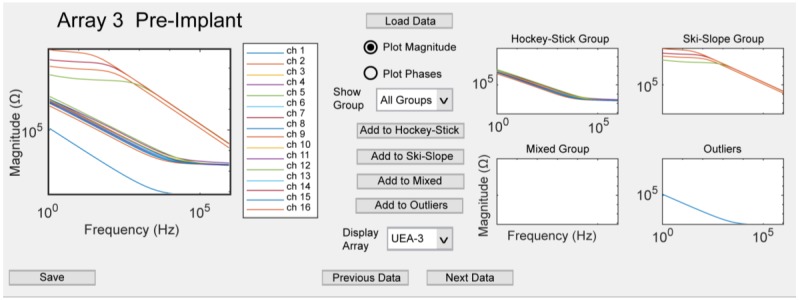
PlotEISGUI with a sample of the iridium oxide Utah electrode array (UEA). In the left plot, the magnitude spectra are plotted for all 16 channels. In the figures on the right, the spectra are divided based on the categories, where Groups 1–3 are the hockey-stick, ski-slope, and mixed groups, respectively.

The magnitude and phase of the impedance was loaded into MATLAB (2017b, Mathworks, Natick, MA, USA) for further analysis. As seen in Figure 2, a custom graphical user interface (GUI), called PlotEISGUI, was designed for the visualization of data (code available, see Supplementary Materials). This was also used to correct any categorization errors that were identified visually.

### 2.4. Categorization Algorithm

**Figure 3:**
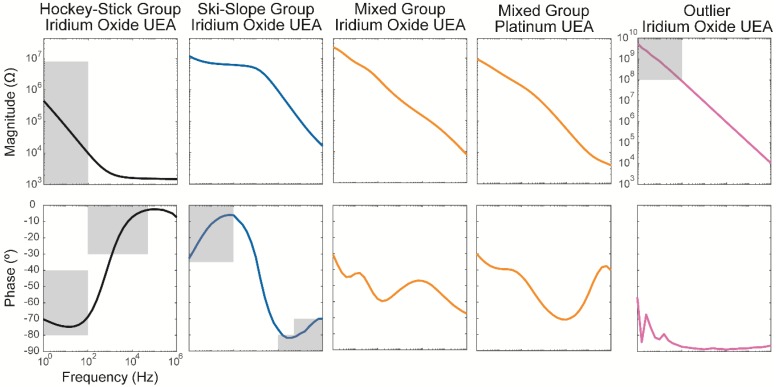
Impedance spectra were classified into groups based on the characteristics of the magnitude and phases. Examples of groups are shown along with categorization criteria (grey boxes; see Section 2.4). Note that the *y*-axis for outliers is scaled to include much higher magnitudes.

A categorization algorithm was developed to separate the spectra for each channel into distinct groups named hockey-stick, ski-slope, mixed, and outliers. A typical example for each group can be seen in Figure 3, along with the inclusion criteria (detailed below) in the shaded boxes. The hockey-stick group has spectra shape common to that of IrOx [4,11,12] and other low impedance materials (e.g., poly(3,4-ethylenedioxythiophene) (PEDOT)), with the magnitude spectra shape being flat at lower impedances occurring at high frequencies. The ski-slope group, named for the shape of the magnitude spectra, which is flat at high impedances occurring at low frequencies. This shape is likely indicative of a parasitic leakage pathway, resulting in a large impedance that is primarily real (and not imaginary), because of the encapsulation damage at the electrode or lead. This also occurs more readily when the electrode impedance is high, because of tip metal degradation or lead wire breakage. The mixed group, with a complex shape with multiple inflections points, is common in all platinum arrays as well as IrOx electrodes after aging and likely associated degradation. The outliers group had very high, often linear impedance spectra that were likely due to site failure or lead-wire breakage in a location, resulting in a higher impedance pathway to the electrolyte.

The default category was a mixed group for both types of arrays. This group consisted a magnitude spectrum with a gradual, consistent slope with no distinct features, and a phase spectrum that had multiple, small inflection points at various frequencies. The intermediate to more negative phases are indicative of a more capacitive character, but these do sometimes have phases closer to 0°, and are associated with more real resistances. The less characteristic fluctuations of this category suggest that it represents a larger diversity of conduction pathways.

For Iridium oxide arrays, some spectra were grouped into a hockey-stick Group. The criteria for the hockey-stick group include all four of the following conditions:

(1) $mean\left(Z_{<100\mathrm{Hz}}\right)<8\times 10^{6}\;\Omega$
(2) $-80^{{}^{\circ}}<mean\left(\varphi_{<100\mathrm{Hz}}\right)<-40^{{}^{\circ}}$
(3) $Max\left(\varphi_{0.1-50\mathrm{kHz}}\right)>-30^{{}^{\circ}}$
(4) $\left|Max\left(\varphi_{0.1-10\mathrm{kHz}}\right)-mean\left(\varphi_{<100\mathrm{Hz}}\right)\right|>25^{{}^{\circ}}$

where *Z* is the impedance magnitude and φ is the phase magnitude, with the mean or maximum value found for frequencies in the subscript. Condition 1 ensured that the impedance at low frequencies was within a typical range, which was lower for a given geometric surface area, because of the use of IrOx. Conditions 2 and 3 evaluated the characteristics of the phase spectrum, where the phase was highly negative at low frequencies and increased with frequency. The phase at lower frequencies is associated with the combination of Faradaic and capacitive characters of the electrodes. In addition, the small phase angle at high frequencies is associated with the access resistance (i.e., the resistance associated with the electrolyte, the path through the electrolyte, the geometry of the electrode, and the geometric surface area of the electrode) behaving more like a pure resistive element. Condition 4 was designed to reflect that a large increase in slope was present, namely, that the phases at low and high frequencies differed more than 25°. This reflects the distinct transition from the spectrum being dominated by the more capacitive electrode–electrolyte interface at low frequencies, to being dominated by the access resistance at high frequencies.

Iridium oxide arrays were also categorized into a ski-slope group, as the magnitude spectrum was flat at low frequencies and decreased at higher frequencies. The phase spectrum was similar to a decreasing sigmoidal function, with small phases at low frequencies and highly negative phases at large frequencies. The criteria for the ski-slope group included the following:

(1) $mean\left(\varphi_{>10\mathrm{kHz}}\right)<-80^{{}^{\circ}}$

or all of the following conditions:

(1) $mean\left(\varphi_{<100\mathrm{Hz}}\right)>-35^{{}^{\circ}}$
(2) $mean\left(\varphi_{>50\mathrm{kHz}}\right)<-70^{{}^{\circ}}$
(3) $max\left(\Delta_{\varphi }\right)>25^{{}^{\circ}}$

where $\Delta_{\varphi }$ refers to the slope of the phase (i.e., the difference in values between successive frequencies). Condition 1 is associated with the highly capacitive behavior that occurs at the higher frequencies for these electrodes; whereas, condition 2 is associated with a relatively high impedance (on the order of 10^7^ Ω) parasitic leakage pathway through the encapsulation. Condition 3 again indicates that a relatively large change in phase angle occurs between low frequencies and higher frequencies, as the impedance transitions from more resistive at low frequencies to capacitive at higher frequencies.

The final group was designated as outliers. For platinum arrays, the channels were categorized as outliers when the average impedance magnitude at <100 Hz exceeded 1 × 10^8^ Ω. For iridium oxide arrays, the channels were categorized as outliers when the average impedance magnitude at <100 Hz exceeded 2 × 10^9^ Ω. The high impedances of these electrodes are at the limits of the impedances that can be accurately measured, and likely represent a failure in the connector system or lead wires near the connector, with the failures having only a high impedance path through the electrolyte associated with failure that is more isolated from the physiological environment.

### 2.5. Equivalence Circuit Model

**Figure 4:**
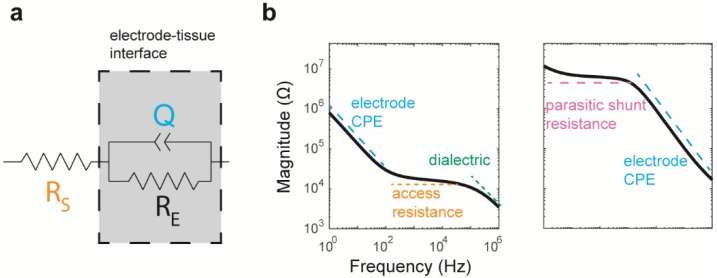
The Randles equivalence circuit used to model an electrode in solution (**a**). The solution resistance, *R_S_*, is in series with the elements denoting the electrode–electrolyte interface, including the electrode transfer resistance, *R_E_*, and the admittance of the constant phase element (CPE), *Q*. Elements have different contributions to the impedance spectra (**b**), as highlighted by examples classified as hockey-stick (**left**) and ski-slope (**right**). In addition to the electrode CPE, *Q*, and access resistance, *R_S_*, parasitic shunt resistance may also be present at low frequencies, as well as coupling through a dielectric capacitance at high frequencies.

The impedance spectra were fitted to a modified Randles equivalence circuit model, seen in Figure 4a [13]. This circuit comprises of an electrolyte resistance *R_s_* (i.e., access resistance), electrode charge transfer resistance *R_E_*, and constant phase element (Z*_CPE_*) representing imperfect capacitance at the electrode/electrolyte interface. Z*_CPE_* is defined as

$$Z_{CPE}\left(w\right)=\;\frac{1}{Q{\left(jw\right)}^{n}},$$

where *Q* is the admittance of the constant phase element and thus a measure of the magnitude, *w* is the frequency, and *n* is a constant between 0 and 1 that describes resistive or capacitive characteristics. The Randles model, which as Z*_CPE_* in parallel with *R_E_*, with these elements in series with *R_S_*. Thus, the equation to fit the impedance spectra is:

$$Z_{EIS}\left(w\right)=\;R_{s}+\frac{R_{E}}{1+R_{E}Q{\left(jw\right)}^{n}}.$$

Each spectrum was fit in MATLAB using Zfit (v1.2.0, 2005, written by Jean-Luc Dellis). The real and imaginary impedances at frequencies of up to 10 kHz were used to create the fit. Higher frequencies were not included in these fits because aging iridium oxide arrays often have a high-frequency roll-off, likely associated with capacitive coupling at a relatively large surface area. This could be from demetallized regions, or areas of silicon exposed by Parylene-C degradation. The initial parameters into the equations were estimated for *R_E_* as 6 × 10^7^ Ω, *Q* as 5 × 10^−7^ S s^−*n*^, *n* as 0.8, and *R_S_* as the real component of the impedance measured at 10 kHz. For the lower and upper bounds, the parameter boundary conditions included 1 × 10^4^–1 × 10^11^ Ω for *R_E_*, 1 × 10^−15^–1 × 10^−5^ for *Q*, 0–1 for *n*, and 10–infinity Ω for *R_S_*.

### 2.6. Machine Learning Algorithm

Using the Classification Learner App in MATLAB, we developed a support vector machine (SVM) algorithm with a fine Gaussian kernel to evaluate the automated categorization for each array type. In this SVM model, the response was the group and predictors included the phase, magnitude, time, and channel for each frequency. Using five-fold validation to protect against overfitting, the algorithm was trained and the prediction accuracy was found.

### 2.7. Statistics

The statistical analyses were performed in MATLAB, with the data reported as mean ± standard error of mean (SEM), unless otherwise noted. The mean and SEM were calculated if more than three channels were present within a specific group at a given time. Unless otherwise specified, the multi-way analysis of variance (ANOVA) was calculated using time, groups, and the log of the impedance magnitude (to normalize values) to detect for significant factors. Significance was determined if *p* < 0.05 after a Bonferroni correction.

To determine predictors for the fit parameters of the equivalent circuit, we created a repeated measures model with the fitrm command in MATLAB. With this repeated measure model, the responses were the fit parameters (*n*, and the logarithm of *R_E_*, *Q*, and *R_S_* to normalize the ranges), and the predictors included the groups, times, and channels. Repeated measures ANOVA (using the MATLAB command ranova) were then used to determine which predictors have a significant effect in the model. Wilcoxon signed-rank tests were then used to compare the parameters between groups, with significance determined if *p* < 0.05 after a Holm–Bonferroni correction.

## 3. Results

The primary goal of the study was to evaluate the longitudinal changes in electrode integrity post implantation. Cohorts of 16-channel Utah arrays were implanted into the sciatic nerve of rats, and the frequency spectra were collected weekly for up to 12 weeks. The implanted arrays were either iridium oxide (*N* = 5) or platinum (*N* = 6) arrays. During the initial analysis, the spectra for each electrode type were averaged to evaluate changes over time. However, it was evident that elucidating trends was problematic because of the large error bars associated with the different spectra shapes that obscured the trends (data not shown). To visualize the individual spectra at specific time points, we developed PlotEISGUI, and observed distinct patterns of the impedance spectra that formed the basis of the categories described below.

### 3.1. Description of Classes

Each site was classified based on select features in the magnitude and phase impedance spectra (see Section 2.4 for details). The electrode sites were categorized into four groups, hockey-stick, ski-slope, mixed, and outliers (Figure 3).

The hockey-stick category spectra look similar to the spectra from the iridium oxide electrodes previously observed in a variety of in vitro studies [4,11,12]. This category was named for the shape of the magnitude spectrum, which has a large negative slope at low frequencies, associated with more capacitive or mixed capacitive, and a Faradaic character that flattened at higher frequencies, behaving like a real resistance. The phase spectrum also had a characteristic shape of either an increasing sigmoid or inverted-U shape, which results from the transition from the dominant impedance transitioning from capacitive/Faradaic to the real impedance. Specifically, a highly negative phase was present at low frequencies, and the phase increased with frequency and either remained high (i.e., closer to zero) or returned to a more negative phase. The hockey-stick group was always associated with iridium oxide arrays for these UEAs and their associated surface area of 4000 µm^2^, including baseline in vitro measurements and some in vivo measurements.

Some of the spectra were categorized in the ski-slope group, where the magnitude spectrum had a consistent, flat slope at low frequencies, which became consistently negative at high frequencies. The Randles equivalence circuit parameters and flat slope of the curve indicate that the electrode was primarily resistive at lower frequencies (see Table 1 and Section 3.3, below). This is likely due to the damage at the wire-bundle lead or to encapsulation, resulting in a parasitic path (Figure 4b), as well as significant tip metal degradation to drive the increased impedance that then reveals the presence of the higher impedance parasitic pathway. The phase spectrum was similar to a decreasing sigmoidal function, with small phases at low frequencies and highly negative phases at large frequencies.

The third group was the mixed group, where the magnitude spectrum decreased with a constant slope and the phase spectrum, and had multiple, small inflection points. For the IrOx arrays, these spectra were similar to one that have been chronically implanted for at least 30 days [11], or to the uncoated sputtered iridium oxide films (SIROF) sites [12], and are thus likely aging sites, with the iridium oxide flaking off, damage to the tips, and/or a complex interaction with the encapsulation sheath. For the platinum arrays, the mixed category was the most prevalent group for all of the timepoints, and the spectra were similar to those previously characterized in vitro [14,15].

Finally, outliers were identified by high impedance magnitudes at low frequencies. These spectra had a very high impedance (see Table 1 and Section 3.3 below), and were likely due to wire failures in the lead that were also well isolated from physiological electrolytes, such as in or near the connector system.

After classification using the automated algorithm, each spectrum was visually assessed using PlotEISGUI. Upon visual inspection, 4.5% (*N* = 35/764) of the classifications were changed for the iridium oxide arrays, with the most common change being from mixed group to outliers (*N* = 32 changes). For the platinum arrays, 3.5% (*N* = 17/1087) of the classifications were adjusted, with mixed group sites being reclassified as ski-slope (*N* = 23) or outliers (*N* = 15). Further analysis was not performed on the hockey-stick or ski-slope groups for the platinum arrays, because of the low number (≤3 channels) of channels at any point of time. For both array types, the algorithm correctly grouped the sites for over 95% of the channels.

**Figure 5:**
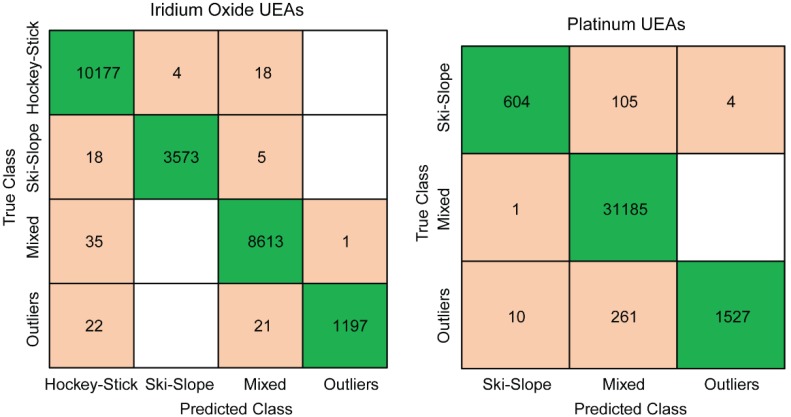
Confusion matrices of the trained SVM algorithm for the iridium oxide (**left**) and platinum arrays (**right**). The numbers refer to the data points within the impedance spectrum, with each spectrum consisting of 31 frequencies collected per channel for each point in time.

To evaluate the strength of the categorization based on the spectra, we employed a machine learning algorithm to determine the power of the classification. We fit a five-fold cross-validation fine-Gaussian SVM to the log of the impedance magnitude, log of the frequency, phase, channel, time, and group. The predictive accuracy is 95% for the IrOx arrays and 98.9% for the Pt arrays, with the confusion matrices as seen in Figure 5. Thus, this machine learning approach is comparable or slightly better (for Pt arrays) than the performance of the categorization algorithm, but at the cost of being more computationally complex.

### 3.2. Longitudinal 1 kHz Impedances

**Figure 6:**
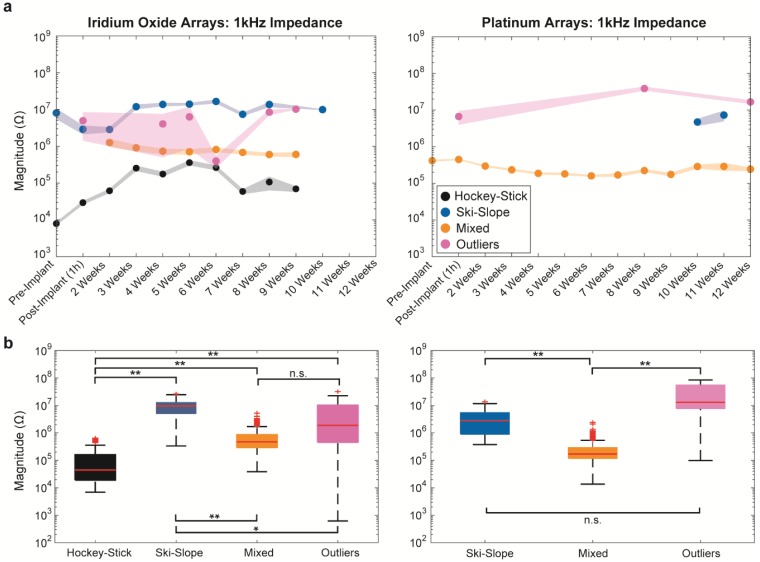
Impedance at 1 kHz for iridium oxide (**left**) and platinum (**right**) arrays vary per group. The mean and standard error of mean (SEM) can be seen at all timepoints (**a**), and the boxplots show the medians for all of the timepoints after implantation (i.e., 1 h through site failure) (**b**) (* refers to *p* < 3 × 10^−4^, and ** *p* < 1 × 10^−13^, after the Holm–Bonferroni correction).

A common method of evaluating electrode integrity is determining the impedance magnitude at 1 kHz. For both the iridium oxide and platinum arrays, we found that groups were a significant predictor of impedances (Kruskal-Wallis, *p* < 1 × 10^−40^), with differences between the groups at all timepoints after the implantation highlighted in Figure 6b. Outliers could not be differentiated from mixed groups for the IrOx arrays, and from the ski-slope group for the Pt arrays, although all of the other group comparisons were significantly different (*p* < 3 × 10^−4^, with Bonferroni correction). Thus, the impedance magnitude at 1 kHz is insufficient to distinguish between all of the groups, and importantly, cannot distinguish between aging, besides the intact electrodes (mixed group) and putative open circuits (outliers) for the IrOx arrays.

Depending on the category, the impedances change in unique ways, as seen in Figure 6a. For the IrOx arrays, the impedances either increase (as in the hockey-stick group) or decrease (as in the ski-slope group) immediately after implantation, in comparison to the impedances measured in vitro. Interestingly, it appears that the impedances for the mixed group of Pt arrays stay similar for 1 h after implantation, compared to the in vitro impedances, although they slowly appear to decrease over time.

### 3.3. Fitting Categories to Randles Model

While the algorithm separated groups based on frequency-specific criteria, fitting the spectra to a Randles equivalent circuit (see Section 2.5 for details) revealed that these differences correlated to the physical properties of the array. For the iridium oxide and platinum arrays, a repeated measured analysis of variance found significant effects for groups (*p* < 2 × 10^−29^), times (*p* < 4.1 × 10^−19^), and channels (*p* < 9 × 10^−5^) (see Section 2.7 for details).

**Figure 7:**
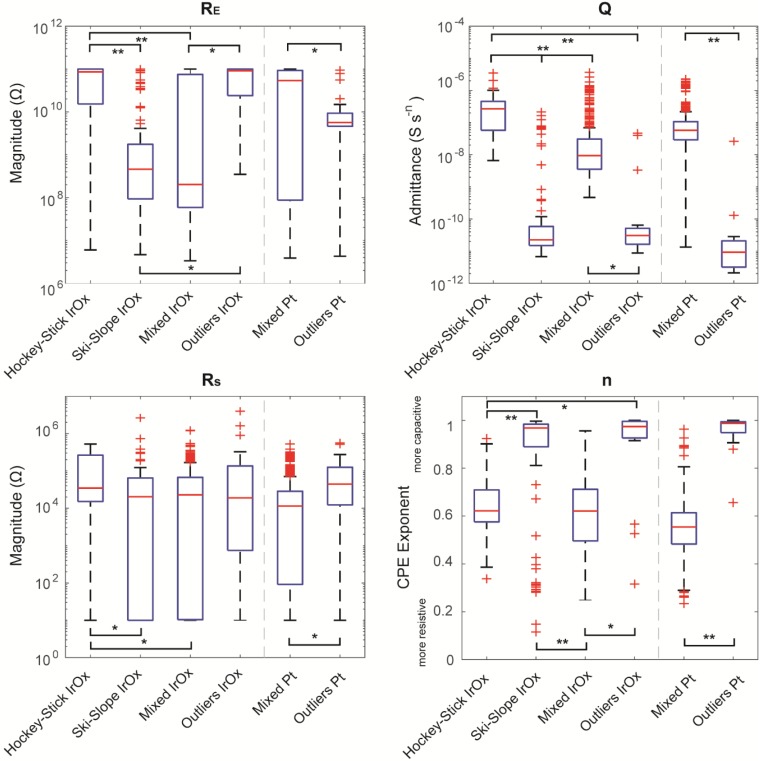
Boxplots of fit Randles equation fit values for in vivo impedance spectra. Boxplots show median values, and comparisons were tested with Wilcoxon sign-rank tests (* refers to *p* < 3 × 10^−4^, ** *p* < 8 × 10^−11^, after a Holm–Bonferroni correction). Note that the upper bound of *R_E_* values were 1 × 10^11^ Ω, as a result of limitations of the Gamry instrument.

Upon inspection, most of the in vitro parameters typically varied dramatically after implantation and then stabilized after the two-week time point. This surgical recovery period is typical to those in previous studies [1,3,16,17], allowing for the encapsulation of electrodes. Thus, we summarized the average fit values for each group for in vitro and in vivo plus two weeks in Table 1, and plotted boxplots of the fit parameters for the in-vivo plus two weeks in Figure 7. The group most reflective of typical IrOx spectra, the hockey-stick group, demonstrated significantly different values for in vivo verses in vitro for the resistance through the electrolyte, *R_S_*; the electrode–tissue interface resistance, *R_E_*; and for the constant phase element, *n*. Similarly, the Pt mixed group, the most prevalent group for this array, had Randles fit equation values that differed for all four parameters (*p* < 2 × 10^−13^) for in vivo in comparison to in vitro. This suggests the critical influence of the in vivo environment on impedance metrics. For both array types, the *R_S_* significantly increased, indicating that either the effective impedance of the electrolyte increased and/or the effective area of the electrode decreased, likely because of biofouling. In addition, both arrays demonstrated a significantly decreased *n* value, indicating that the electrode was acting more resistive, potentially because of an increase in the effective area. For the Pt arrays, the constant phase element admittance, *Q*, value significantly increased, likely because of the decrease of impedance, especially at low frequencies. For the IrOx arrays, there was no significant difference with the *Q* value over time, although the distribution of values became increasingly skewed with time (data not shown), indicating that the mean is less representative of the population. Finally, both the *R_E_* values increased for both of the arrays, although this is likely from noise, because some in vivo values matched the upper limitations of the model, set at 1 × 10^11^ Ω to mimic the measurement limitations of the Gamry.

Next, we wanted to determine whether these Randles circuit parameters reflect the differences between groups by determining whether comparisons are significant. For the in vitro spectra of the iridium oxide arrays, the hockey-stick group had a significantly different *Q* value than the ski-slope group (*p* < 0.003, Wilcoxon signed-rank tests with Holm–Bonferroni correction). In addition, the hockey-stick group had a different in vitro *R_S_* from the mixed group (*p* < 0.003). The differences between the groups became more prevalent when looking at the plus two weeks in vivo values. The hockey-stick group was different from the ski-slope for all four of the parameters (*p* < 4 × 10^−6^); different from the mixed group for *R_E_*, *Q*, and *R_S_* (*p* < 2 × 10^−9^); and different from the outliers for *Q* and *n* (*p* < 2 × 10^−5^). In addition, the ski-slope group had different values for *Q* and *n* than the mixed group (*p* < 3 × 10^−24^) and a different *R_E_* value from the outliers (*p* < 3 × 10^−7^). Finally, the mixed group had significantly different *R_E_*, *Q*, and *n* values than the outlier group (*p* < 3 × 10^−4^). For the Pt arrays implanted for at least two weeks, the values for the mixed group were all significantly different than the outliers (*p* < 8 × 10^−5^). Together, this demonstrates that the groups differ because of the physical features at the electrode and electrode–electrolyte interface.

The Randles circuit *n*-value, which represents how capacitive or resistive the electrode is behaving, had stark differentiation between groups. By definition, the *n*-values range from 0 to 1, depending on whether the constant phase element in the Randles circuit behaves more like a capacitor or resistor, respectively. For the chronically implanted iridium oxide arrays, the hockey-stick and mixed groups had *n*-values that averaged approximately 0.6, indicative of a leaky capacitor common to that of IrOx. In contrast, the ski-slope and outliers for both of the array types had much higher *n*-values of 0.85 or more, indicating a leakage path of a shunt resistor. This is likely due to breakage in the wire bundle, allowing for the current to flow directly to the environment. For the ski-slope group, this is also demonstrated by the lower *R_E_* values, indicative of a greater charge transfer to tissue.

The constant phase element admittance, *Q*, also greatly varied between groups. The hockey-stick groups had the highest values at approximately 30 × 10^−8^ S s^−*n*^, with the mixed groups having slightly lower values at 11 × 10^−8^ S s^−*n*^. In contrast, the groups that likely demonstrate damaged sites/wire bundle, the ski-slope and outlier groups, have values of 1 × 10^−8^ S s^−*n*^ or lower.

The shunt resistance, *R_E_*, also reveals differences between the categories, with the exception of the hockey-stick to outliers, and ski-slope to mixed. However, this may not be representative, because the value is near or at the upper bound of 10^11^ Ω defined in the Randles model, a limitation incorporated because this is the limit of the impedance that the spectrometer can measure. In addition, this may further be limited, because we did not collect frequencies lower than 1 Hz. Collecting impedances at lower frequencies may illuminate further difference between the categories.

The access resistance, *R_S_*, is associated with the effective impedance of the tissue/electrolyte between the electrode and reference, as well as the effective geometry and geometric surface area of the electrode, and is often observed to increase during in vitro and in vivo studies as a function of tissue encapsulation or fouling (e.g., [18]). The increases in impedance can also occur as metallization is lost, and thus the effective electrode area is decreased. Because of the particularly large difference in impedance between silicon and IrOx, this value can be sensitive to the loss of metallization with resulting exposure of Si, which has been reported [4]. It also depends on the effective conductance of the physiological environment, which can be modulated by the types of tissue or the foreign-body response associated with the electrode. For mixed and outlier groups, there is no flat region of the spectra associated with the access resistance, indicating that the categories are always limited by the impedance of the electrode/electrolyte interface, where the “electrode” might be a broken, but it encapsulates the wire in the lead, particularly for the case of the outliers.

The Randles model also highlights the differences between the types of arrays. Comparing the values between the hockey-stick group and the mixed Pt group (i.e., the channels most like typical IrOx and Pt sites), all of the plus two week in vivo fit values were significantly less for the Pt sites (*p* < 1 × 10^−10^, Wilcoxon sign-rank tests). In addition, the *R_E_*, *Q*, and *R_S_* values were also lower in vitro for the Pt mixed group (*p* < 0.005). Given that higher *n*-values indicate that the model is acting more as a capacitor, it is unexpected for the *n*-values to be lower for the mixed Pt group. This may be due to the *n*-values for the Pt mixed group being lower here than in the previously published in vitro results [1], although it is important to note that these values are from planar electrodes. However, given that IrOx is known to have lower impedances than Pt sites, it is unsurprising that the values are lower for *Q*, with lower Q values being consistent with a lower impedance magnitude of the constant phase element.

### 3.4. Longitudinal Changes of Categories

**Figure 8:**
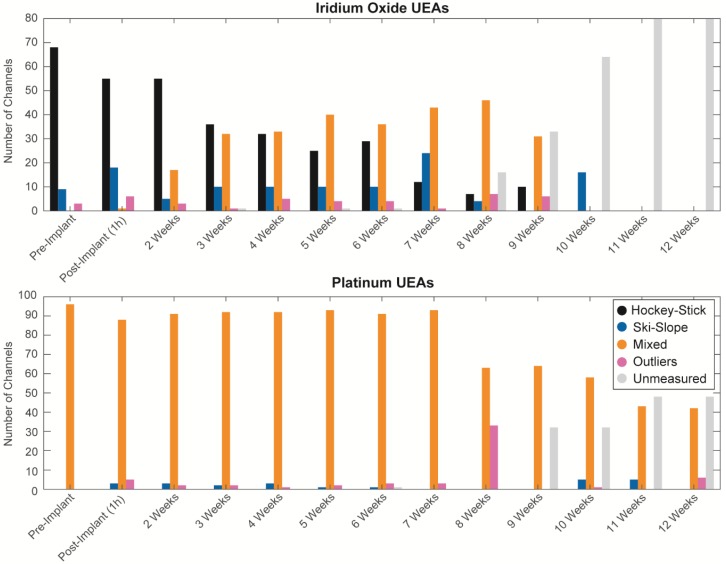
Summary of population changes for groups over time for iridium oxide (**top**) and platinum (**bottom**) arrays. After the failure of all sites in an array, the impedance spectra were no longer collected and are designated as unmeasured.

The distribution of groups changes over time, as seen in Figure 8. For the iridium oxide arrays, the sites are primarily hockey-stick in shape, until three weeks post-implantation. The mixed groups become more prevalent starting at two weeks, and continuously increase through to week eight. The spectra for the IrOx mixed group appear similar to the IrOx arrays implanted for at least 30 days, tested by Cogan et al. [11]; the Pt electrodes from this and other UEA studies; uncoated SIROF (i.e., just the gold metallization for planar electrodes) sites [12]; and the analysis of impedance as a function of the materials and surface area [4]. The lower impedance expected for IrOx suggests that the transition from the hockey-stick to mixed arrays is likely attributable to a loss of surface area resulting from IrOx delamination and/or fouling associated with adsorption and glial encapsulation during aging. This results in the impedances remaining above the access resistance associated with the flat part of the hockey-stick curve only potentially occurring at higher frequencies (e.g., Figure 4b). Starting at week eight, many sites stop being measured because of device failure, with the last sites being measured at week ten. For the Pt arrays, the mixed group is the predominant group until week 11, at which point 50% of the sites have failed. Overall, there are few sites that belong to other groups until week eight, when over 30 sites become outliers. Thus, the Pt arrays demonstrate more stable impedance spectra than the IrOx arrays. This is possibly, in part, due to the higher starting impedance values for the Pt electrodes, decreasing the changes in impedance associated with metal delamination. A careful SEM evaluation of the metal degradation would be required to discriminate these.

**Figure 9:**
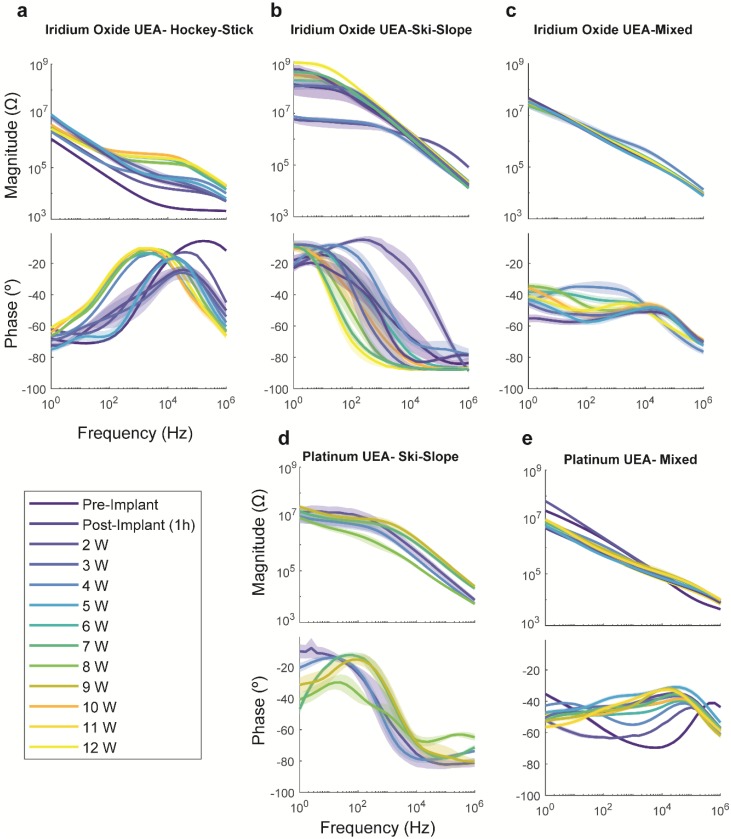
Mean magnitude and phase spectra for iridium oxide (**a**–**c**) and platinum (**d**,**e**) UEAs (shaded areas are SEM) over time.

Within a group, the impedance spectra change over time in complex ways, as seen in Figure 9. For the iridium oxide arrays, the hockey-stick group has a phase spectrum that initially looks like an increasing sigmoid function (dark blue in Figure 9a, bottom panel). After implantation, the peak phase shifts to lower frequencies and forms a maximum at approximately 1 kHz, by approximately nine weeks (yellow shades in Figure 9a). As time progresses, the curve maximum occurs at incrementally lower frequencies. This maximum is associated with phase angles closer to zero, and therefore resistive impedance character. When associated with the magnitude spectra, an incrementally increasing resistance for the flat part of the curve is observed, which we associate with the access resistance. This increase in resistance causes this component of the curve to intersect the sloped region at lower frequencies, resulting in the concomitant propagation of the maximum to lower frequencies. The results are consistent with an increasing impedance for the physiological electrolyte or a decreased surface area for the electrode. These could be associated with the foreign body response and tissue healing process, and tip metal degradation, respectively. For each hockey-stick spectrum, we found f_peak_, the frequency at which the maximum phase occurred, and found that time has a significant effect (*p* < 7 × 10^−49^, Kruskal–Wallis test). Using Wilcoxon signed-rank tests to compare across timepoints, we found the f_peak_ to be progressively lower up to week six. After approximately six weeks, this f_peak_ value becomes similar. This phase peak is reflected in the magnitude spectrum via the high-frequency roll-off (i.e., the elevated magnitude at approximately 10 kHz). This roll-off is thought to be due to the degradation of the Parylene-C and current leakage through the silicon or even capacitive coupling through the dielectric [4] (see Figure 4b).

For the iridium oxide arrays belonging in the Ski-Slope group (Figure 9b), the phase spectrum appears as a decreasing sigmoid. Immediately after implantation, the inflection point of this sigmoid (i.e., greatest slope) occurs at a higher frequency. In the successive weeks, the inflection point occurs at progressively lower frequencies, without statistical significance (*p* > 0.05).

For the platinum arrays, the phase spectrum for the predominant mixed group initially presented with a U-shaped minimum in middle frequencies, similar to that of Pt UEAs previously shown in vitro [14,15]. As time progresses, this minimum flattens out and a high-frequency peak develops. When similar electrodes age in a saline bath, the size of this minimum decreases, although it does not become as flat as those observed here at approximately seven weeks. Thus, these changes are likely due the foreign body response, although the aging of the electrode may also contribute. The magnitude spectra are similar [14,15] or much lower than a previous study [19], with the latter difference potentially arising from the large error bars presented with the mean. Over time, we found that the impedance decreased at low frequencies (<100 Hz) and increased at very high frequencies (1 MHz). Electrodes aging in a PBS bath decreased in impedance at low frequencies, but remained similar at high frequencies [14]. In contrast, reactive accelerated aging via exposure to reactive oxygen species decreased the impedance across all of the frequencies [19]. These differences are likely attributed to factors arising from glial response in vivo.

## 4. Discussion

We developed a simple categorization algorithm for the impedance spectra that can enable a more thorough longitudinal analysis of the implanted electrodes. We also created a visualization GUI, called PlotEISGUI, to display the categories and enable corrections with visual inspection. We found this algorithm to be robust and it correctly categorized 95% of the spectra, with correction required for approximately 5% of the spectra. This performance was comparable to a fine-Gaussian SVM algorithm, which had a prediction accuracy of 95% for iridium oxide arrays and 98.9% for platinum arrays. The advantages of the algorithm over the SVM included its simplicity, which allows it to classify an individual electrode’s spectra at one point in time with computation efficiency and without expensive toolboxes. The groups are further distinguished by the difference in fit parameters when modeling each spectrum to a Randles equivalence circuit, further demonstrating the distinctiveness of each category.

### 4.1. Physical Characteristics Underlying Groups

The hockey-stick shape described here is common to the spectra previously observed in the iridium oxide arrays [4,11,12]. As the IrOx electrodes age, the hockey-stick group develops a characteristic flattening, with an increased impedance with time at approximately 100 Hz–10 kHz, similar to the previously observed spectra of the implanted electrodes [16]. This flat aspect is associated with the access resistance, which is controlled by the geometric surface area of the electrode, the geometry of the electrode (including the length), and the effective impedance of the path through the (physiological) electrolyte (e.g., Figure 4b). The impedance of the physiological environment can change because of the formation of the glial scar around the electrodes, or other tissue healing or responses for the tissue between the electrodes. In unpublished studies, we have observed that this increase in access resistance begins within minutes after implantation. This effect is not observed for the Pt arrays, which actually have a small yet significant decrease in 1 kHz impedance after implantation (comparing in vitro to week three and following time points, *p* < 1 × 10^−6^ after Bonferroni correction). The Pt impedance is higher than IrOx, and typically has a smoother surface, making increases in impedance with in-dwelling time smaller. Also, implantation can decrease the impedances through encapsulation degradation and water ingress. Additionally, Pt microelectrodes, with their associated small surface area (~4000 µm^2^), do not demonstrate a flat region associated with the access resistance. Comparing the in vitro values of the Randles fit equations to those of the IrOx arrays found by Caldwell et al. [4], the latter reported a *n* of 0.87, which is within one standard deviation of the value presented here. They also reported Q_0_ of 27 × 10^−6^ S s^−*n*^ mm^−2^, which when multiplied by our size electrode (i.e., 4000 µm^2^), would be equivalent to 1.1 × 10^−7^ S s^−*n*^, a value approximately one standard deviation from our mean of 2.2 × 10^−7^ S s^−*n*^. In addition, aging electrodes in the hockey-stick group also developed a high-frequency roll-off, which is thought to be associated with leakage through the dielectric capacitance [2].

The ski-slope group for IrOx is likely a result of lead wire breakage or a significant loss of tip metallization, and the presence of a parasitic leakage path responsible for the flat region at low frequencies (e.g., Figure 4b). The parasitic leakage path often occurs at very high impedances of 10^8^ to 10^9^ Ω, and therefore can only be observed when the impedance of the channel is high enough that it can be appreciated. The individual gold lead wires are overmolded by silicone; therefore, when these wires break, their impedance can increase modestly if the break is in a way that results in a lower impedance path, or with a significantly higher impedance because of poor communication to the electrolyte. The flat region at low frequencies has a resistive character, suggesting that this is a small/high impedance leakage path, potentially associated with encapsulation degradation. This is supported by Randles circuit models, where the in vivo *n*-values are 0.85, and the *R_E_* values are below that of the other groups, indicative of a leakage path due to a shunt resistance and greater charge transfer to tissue. The increase in *R_E_* values suggests that either tissue remodeling or further material degradation results in an increased impedance for the shunt pathway. The mechanism of wire breakage is further supported by the fact changes in group categorization to the ski-slope group were often global across the array. Specifically, for two IrOx arrays, at least 15 of the 16 of sites became designated as ski-slope at the same point in time.

For the iridium oxide arrays, the mixed group interpretation of the impedance spectra is more difficult and would benefit from the physical characterization of the electrodes using SEM, optical microscopy, and other failure analysis techniques. The impedance magnitudes are similar to the hockey-stick group at high frequencies (>10^5^ Hz, especially for hockey-stick sites after approximately six weeks), but is ~2 to 10 times higher than the hockey-stick sites at 1 Hz. The higher impedances at low frequencies for the mixed group electrodes result in *R_S_* not dominating the impedance spectra at intermediate or high frequencies. In addition, there is a large difference in the phase curves between these two groups. The phase curve for the hockey-stick indicates a relatively capacitive coupling at low frequencies, because of more negative phase values, which increase towards values closer to zero, indicative of a resistive character. In contrast, the mixed electrodes maintain a relatively consistent phase angle across the spectra from low to intermediate values. This is similar in character and magnitude to the spectra from the Pt electrodes. The loss of the flat region characteristic of the hockey-stick spectrum indicates that the impedance of the electrode interface dominates the spectra across the measured range. This can result from a loss of geometric surface area for the metallization, or a decrease in effective capacitance, resulting in an increased impedance. The former would result from metal delamination, or the effective surface area being decreased by the foreign body response. The increase in effective capacitance could result from a change in the surface material; to the underlayers of Pt and Ir if the IrOx delaminates, leaving those layers behind; or even the silicon surface of the shank. In principle, fouling of the IrOx surface could also result in a decreased capacitance, but this is at odds with the behavior of the aged IrOx that maintains the hockey-stick character.

The platinum arrays were most commonly assigned as the mixed group. Compared to the Pt UEAs in saline, these spectra were similar [14,15] or lower [19] in impedance to those previously observed. For the data with a higher impedance, the study found large error bars when averaging across the 16 channels [19], which makes interpretations more challenging. In addition, the spectra summarized here were similar in magnitude but not phase to planar black Pt microelectrode arrays with a tip size of 900 µm^2^ [1]. For the planar electrodes, the Randles equivalence circuit parameters were as follows: *Q* was 0.89 ± 0.52 × 10^−9^ sΩ^−1/*n*^, *n* was 0.86 ± 0.02, *R_S_* was 7.38 ± 1.74 kΩ, and *R_E_* was 2.7 ± 1.31 × 10^9^ Ω (mean ± standard deviation). The *R_E_* and *n* values were similar to the ones presented here, at approximately 0.1 and 1.4 standard deviations from our determined values, respectively. Given that *R_S_* reflects the size of the electrode, we divided this value by the ratio of sizes, assuming that our Pt UEAs were approximately 4000 µm^2^. This results in a corrected mean of 1.7 kΩ, which is approximately half a standard deviation from the mean found here. To compare *Q* values, we converted the units by multiplying to the power of *n*, with a value for *Q* of 1.6 × 10^−8^ (S s^−*n*^), which is approximately 1.5 standard deviations of the mean values presented here. The mixed group for the Pt array also showed a pronounced phase shift over time, with a high-frequency maxima developing several weeks post implantation. This is consistent with previous work suggesting the development of moderate encapsulation around Utah electrode arrays implanted in rodent cortex [20].

The ski-slope group for the Pt electrodes was different than for IrOx, in particular, with lower impedances for the flat aspect of the spectra. This suggests that the impedance associated with the parasitic shunt pathway had a lower impedance (Figure 4b), and therefore had a large area. This suggests a greater degree of encapsulation degradation for the electrodes, as a roll-off at low frequencies has been consistently found as an early hallmark for this degradation across both test structures and for electrodes.

### 4.2. Categorization Algorithms

The elegance of this approach allows for the differential categorization of electrodes within a given array—providing electrode-by-electrode information on interface dynamics. This categorization can be accomplished with a simple equation, and does not require large datasets, complicated machine learning algorithms, or expensive toolboxes. Importantly, this approach allows for the improved characterization of array dynamics over time. The dual inclusion of magnitude and phase characteristics can separate resistive and capacitive evolution over the course of in vivo implantation.

Interestingly, the categorization criteria relied heavily on the phase values, and supplemental on impedance magnitude at frequencies <100 Hz. This is consistent with the data from in vitro accelerated aging studies incorporating reactive species, which demonstrated that the largest changes in the aged electrodes were in frequencies <100 Hz, regardless of the initial electrode materials, form factor, or impedance spectra characteristics [19].

In contrast, the information at 1 kHz, as commonly used to describe the electrodes, was less informative. Impedance at 1 kHz could not differentiate between the outlier and mixed groups for the IrOx arrays, and between the outlier and ski-slope groups for the Pt arrays, with the outlier groups likely being broken electrodes. In addition, the 1 kHz measurement showed little temporal variation, and stayed relatively similar between the in vivo and in vitro measurement environments. Interestingly, we did not observe marked increases in impedance at 1 kHz in the first several weeks post-implantation, in contrast with other measurements of devices implanted in the central nervous system [2,5,17,21,22,23]. This may be a feature of implantation in the peripheral nerve stimulation (PNS) or, the implantation method using a silicone cuff and fibrin sealant, which may change the immediate physiological environment and itself be degraded over time. Alternately, this may suggest minimal edema following implantation, as transient increases in impedance have been tied to transient biofouling, edema, and neuroinflammation in the central nervous system (CNS) [23,24,25].

However, the evaluation of the full spectrum EIS showed robust temporal dynamics over the course of implantation. This more accurately matches what is known regarding the time-evolving tissue response [26,27,28,29,30], and the gradual degradation in electrode materials known to occur in vivo [17,31]. For instance, the population of the hockey-stick group declined following three weeks in vivo, representing dynamic changes to the electrodes following implantation. These changes likely result from increased impedances associated with metal delamination or from significant fouling of the IrOx, possibly counterbalanced by water penetration through the Parylene-C dielectric [19,32], as evidenced by the roll-off seen at high frequencies (e.g., Figure 4b).

Anecdotally, we found that changes in the assignment of categorization for an individual electrode was often reflective of changes throughout the array. For one iridium oxide array, 15 of 16 electrodes were designated as hockey-stick before implantation. During implantation, one electrode became an outlier, and the remaining 14 were categorized as hockey-stick until week eight, when all of the sites became Ski-Slope. The following week, the array was no longer measured because of failure. A similar change happened for another IrOx array, when at 1 h post implantation, 15 sites were hockey-stick, and all 15 sites became mixed group at week three. The sites remained at the mixed group until array failure at week 10. A third array was more variable, all of the electrodes were hockey-stick both in-vitro and immediately post implantation, and at two weeks, 14 sites were hockey-stick and two sites were mixed group. By week five, three sites were outliers, five sites were mixed, and the eight remained hockey-stick. This remained varied until week 10, when all of the sites became hockey-stick, regardless of their previous category. For the remaining two iridium oxide arrays, the sites were more varied in the categories from the in-vitro tests. Platinum arrays similarly have universal changes to sites. For two platinum arrays, at least 15 sites were in a mixed group until week eight, when all of the sites became outliers. In another two arrays, at least 15 sites were mixed until week 12. Perhaps these global changes result from abiotic factors such as damage to lead wires, delamination at the bond pad, connectors, or abrupt biological changes, such as bleeding.

### 4.3. Future Work

The data presented here analyzes the changes to the impedance spectra over time, creating categories to allow for the analysis of trends over time. It will be critical to correlate those changes with the physical changes of the electrode tips. At the completion of the study, the arrays were harvested and imaged using scanning electrode microscopy (data not presented here). The results will further illuminate whether these categories correlate to specific physical changes or damage to the electrode.

This work shows changes in electrode spectra when implanted chronically in the peripheral nerve of a rat. It will be informative to determine whether similar effects are found for similar electrodes implanted in the central nervous system, as well as within different animals. In addition, collecting spectra from a wider range, including frequencies down to 0.01 Hz, may provide further information about nonlinear effects at low frequencies. For iridium oxide electrodes, relating cyclic voltammetry to the different groups may provide further information about the charge density and health of the site. 

## 5. Conclusions

These data represent a new approach to characterizing broad spectrum EIS from electrodes implanted in vivo. Utilizing EIS provides meaningful information that cannot be conveyed with a single frequency impedance measurement. Critically, a 1 kHz impedance was not able to differentiate between functional and presumed broken channels. We found that the key characteristics for defining the variability of in vivo electrodes were shifts in the phase and impedance magnitude at <100 Hz. The classification algorithm that we developed is a rapid, resource-efficient classification tool to evaluate longitudinal, broad spectrum EIS data. The application of this algorithm to Utah arrays implanted in the rat sciatic nerve demonstrated that iridium oxide electrodes rapidly alter their properties in vivo, while the platinum arrays had greater stability. This difference may be due to the lower impedance of the iridium oxide sites, which make the changes in aging arrays more easily observed, in contrast to platinum, which has a more similar impedance to Silicon, and thus the changes could be more difficult to elucidate. The relatively low impedances of the IrOx, and the fact that the access resistance is the limiting resistance for these, might result in the aging of the electrodes being more easily observed. In addition, abrupt categorization changes often preceded array failure, and seemed to implicate lead wire breakage as a major failure mechanism. Future work will relate these classifications to alterations in the physical properties of the device materials and to the quality of the detected neural signal.

## Figures and Tables

**Table 1:** Summary of the circuit parameters’ fits to Randles equations. The mean +/− standard error of mean (SEM) of each variable is shown both in-vivo and in-vivo past two weeks implantation. No outliers were present for in-vitro conditions for either array.

Array Type	Group	Condition	Number Spectra	*R_E_* (10^10^ Ω)	*Q* (10^−8^ S s^−*n*^)	*n*	*R_S_* (kΩ)
Iridium Oxide	Hockey-stick	In vitro	67	2.0 ± 0.4	22 ± 1	0.81 ± 0.01	2.17 ± 0.07
		In vivo	205	6.2± 0.3	30 ± 2	0.64 ± 0.01	138 ± 12
	Ski-slope	In vitro	9	2.1 ± 1.2	7.4 ± 3.2	0.68 ± 0.11	156 ± 90
		In vivo	99	1.0 ± 0.3	1.1 ± 0.3	0.85 ± 0.02	76 ± 28
	Mixed	In vitro	4	4.5 ± 2.6	43 ± 34	0.75 ± 0.09	0.15 ± 0.12
		In vivo	299	3.0 ± 0.2	11 ± 2	0.61 ± 0.01	59 ± 7
	Outliers	In vitro	0				
		In vivo	17	6.8 ± 1.0	0.53± 0.35	0.89 ± 0.05	416 ± 245
Platinum	Mixed	In vitro	96	0.46 ± 0.18	0.72 ± 0.08	0.74 ± 0.01	7.3 ± 1.3
		In vivo	854	5.0 ± 0.1	11 ± 1	0.55 ± 0.01	25 ± 2
	Outliers	In vitro	0				
		In vivo	42	1.1 ± 0.3	0.064 ± 0.062	0.96 ± 0.01	93 ± 20

## Supplementary Materials

The code for PlotsEISGUI and the categorization algorithm are available online at: https://bitbucket.org/MargoS/eis-analysis.git.
